# Two Cases of COVID-19-Related Deaths Unaccounted for: A Call for Action

**DOI:** 10.7759/cureus.26238

**Published:** 2022-06-23

**Authors:** Osamu Usami

**Affiliations:** 1 Department of Respiratory Medicine, Kurihara Central Hospital, Miyagi, JPN

**Keywords:** pneumonia, mortality, rehabilitation, pcr, sars-cov-2, covid-19

## Abstract

Coronavirus disease 2019 (COVID-19) high-risk survivors experience long-term COVID-19 symptoms. Hence, these individuals require early and ubiquitous respiratory rehabilitation to avoid malnutrition. We report the case of a 93-year-old woman who recovered from moderate II severity (pneumonia requiring oxygen). The patient, after prolonged hospitalization, demonstrated low severe acute respiratory syndrome coronavirus 2 (SARS-CoV-2) infectivity and showed no COVID-19 respiratory symptoms for more than 72 hours. Subsequently, the patient became debilitated and lost her appetite without dysphagia, dysgeusia, and smell disorder, developed nosocomial pneumonia as a sequela of acute COVID-19 and died. We also report the second case of an 84-year-old man diagnosed with moderate II COVID-19 severity. After recovery, the patient was frail due to the previous onset of COVID-19 and worsened during his stay at home, losing appetite without dysphagia, dysgeusia, and smell disorder, and dying of senility as the official cause. Recovered COVID-19 appears to be a health risk by malnutrition without anorexia and depression, among other conditions. A proven rehabilitation program for each phase of the disease is required for better lung function and nutritional status.

## Introduction

Severe acute respiratory syndrome coronavirus 2 (SARS-CoV-2) infection has overwhelmed the world. The mortality rate of coronavirus disease 2019 (COVID-19) is lower than that of Severe acute respiratory syndrome (SARS) or Middle East respiratory syndrome coronavirus (MERS-CoV). In contrast to the influenza virus, SARS-CoV-2 has infectivity before symptoms arise in addition to clinical presentation, and symptoms are more severe [1]. Asymptomatic infectious cases spread around public spaces. The effective prevention measures practiced thus far include social distancing, hand hygiene implementation, and vaccination. Several COVID-19 waves identified high-risk factors, such as high body mass index, advanced age, and immunocompromised history. These high-risk survivors experience long-term COVID-19 symptoms, including low activities of daily living (ADL), deteriorating lung function, and malnutrition. Moreover, diffuse alveolar-damaged COVID-19 lungs are not easily recovered; thus, early and ubiquitous respiratory rehabilitation is required because there are several COVID-19 cases with damaged lungs [2]. I investigated how researchers consider malnutrition as a COVID-19-related sequela.

Here, I describe two low ADL COVID-19-related deaths due to appetite loss without dysphagia, dysgeusia, ageusia, infectivity, and cytokine storm formation.

## Case presentation

First case

**Figure 1:**
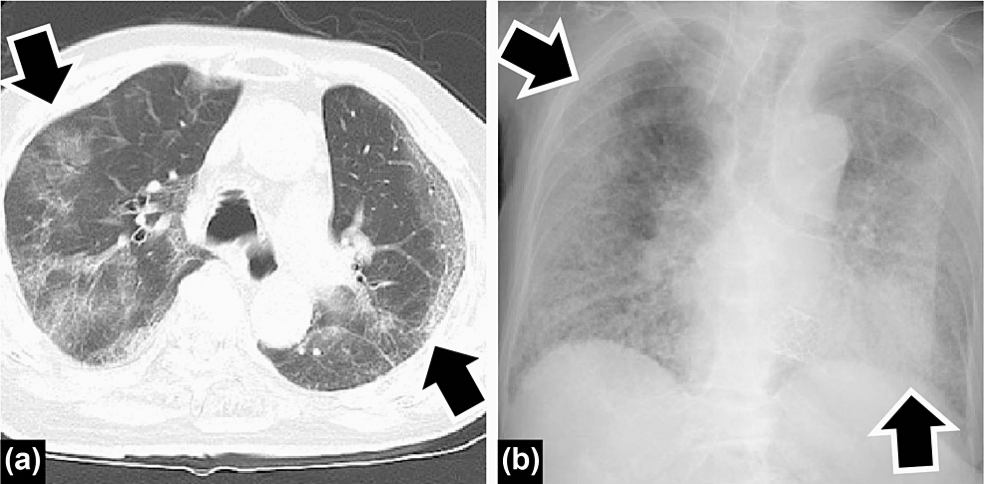
Chest images of the first patient (a) Chest computed tomography on admission. Peripheral predominant ground-glass shadows present in bilateral lung fields; (b) Chest radiography at secondary pneumonia. Diffuse infiltration shadows in bilateral lung fields.

A 93-year-old woman was exposed to a nursing home COVID-19 cluster. She had a history of hypertension, heart failure, and aortic valve stenosis. Her body temperature (BT) increased to 38 °C on April 17, 2021. After the patient took acetaminophen several times, her BT decreased to 37 °C 3 days after COVID-19 onset. On April 19, 2021, the patient’s SARS-CoV-2 antigen test was positive. Her SARS-CoV-2 polymerase chain reaction (PCR) test was also positive the next day. Upon admission to our hospital, the patient’s clinical status was as follows: oxygen saturation (room air), 86%; lactate dehydrogenase (LDH), 504 U/L; C-reactive protein (CRP), 11.2 mg/dL; and procalcitonin, 0.34 ng/mL. Her chest computed tomography image was compatible with COVID-19 (Figure 1a), and she was diagnosed with moderate II COVID-19 severity (pneumonia requiring oxygen) as Japanese severity criteria [3]. Mechanical ventilation as a treatment option was discussed with her family because our hospital did not have mechanical ventilation support for COVID-19. The patient’s family agreed to oxygen administration using a non-rebreather mask preferring it over invasive therapy. The patient’s oxygen saturation increased to 92% (using a 4-L mask), and from the next day of admission, the patient received remdesivir for five days and dexamethasone (6.6 mg per day for 10 days), based on our hospital protocol for moderate II severity disease, to suppress diffused alveoli damage and cytokine storm caused by the virus. Atrial fibrillation was noted, and bisoprolol was started to control the patient’s heart rate. Some studies reported the incidence of venous thromboembolism by autopsy in patients receiving bisoprolol, but the patient, in this case, did not receive anticoagulants. There was no evidence that the benefits outweighed the risks at that time, and even low-molecular-weight heparin was considered off-label use [4]. Because the patient had no appetite, she was under fasting and received fluid therapy.

At the end of April, her BT returned to normal (37.0 °C), antibiotics were discontinued, and no pathogenic bacteria were detected in the sputum. Based on health center criteria, our hospital protocol considered the patient as not having infection anymore as no COVID-19 respiratory symptoms were registered for more than 72 hours; moreover, her Charlson comorbidity index was 2 (medium). The patient resumed eating and had no dysphagia, dysgeusia, or smell disorder, but gradually she had loss of appetite again; although she was bedridden, respiratory rehabilitation was not possible owing to budget issues. In May, attempts were made to transfer the patient to several supporting hospitals; however, the target hospitals declined the patient because they did not have a negative-pressure isolation room or already had full occupancy. On June 1, 2021, the patient’s BT increased again. Because more than 10 days had passed after the patient’s COVID-19 onset, it was assumed that her SARS-CoV-2 infection was diminished and that her increased BT was due to COVID-19 secondary pneumonia (Figure 1b). The potential diagnoses were aspiration pneumonia, organizing pneumonia, and drug-induced interstitial pneumonia. The possibility of aspiration pneumonia was low because her appetite was poor and a high-calorie infusion from the central vein was administered in a fasting state. Moreover, organizing pneumonia after COVID-19 is rare [5]. Finally, interstitial pneumonia could not be excluded even if she did not experience diffused alveolar damage [6]. Therefore, the patient was thought to have experienced lung dysfunction by SARS-CoV-2 infection. Because the patient had negative SARS-CoV-2 PCR results on June 10 and 11, her transfer to a supporting hospital was stopped and she was transferred to our general intensive care unit (ICU) instead. Two negative PCR results were convincing for the ICU staff. The patient’s blood and sputum cultures were negative. After ceftriaxone (1 g q12h) was administered, as our antibiotic stewardship team suggested, ceftriaxone was changed to meropenem (1 g q8h). Her general condition worsened despite providing intensive supportive care and antibiotics, and she experienced disseminated intravascular coagulation. She subsequently died on June 21, 2021. The cause of death was secondary pneumonia induced by lung dysfunction as a long-term COVID-19 symptom. The patient did not spread SARS-CoV-2 in the general ward or ICU and did not receive physical rehabilitation for breathing or swallowing training.

Second case

**Figure 2:**
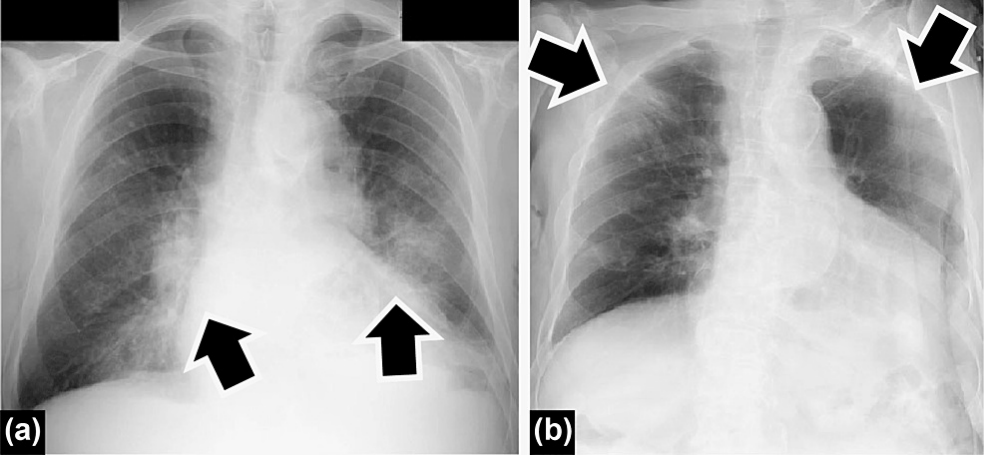
Chest radiography images of the patient in the second case (a) At revisit. (b) At the general ward.

An 84-year-old man had a history of hypertension and atrial fibrillation, and he took only antihypertensive medications daily, and his Smoking Brinkman Index was 200. The patient experienced fatigue on August 14, 2021, and visited our hospital on August 16. Subsequently, SARS-CoV-2 antigen result was positive, but chest radiography did not reveal any sign of pneumonia. The patient did not complain of any dyspnea or other respiratory symptoms and had a strong appetite; therefore, the health center categorized him in the mild group (symptoms without pneumonia and hypoxia), and stayed at home for home isolation. On August 21, the patient revisited our hospital complaining of shortness of breath and appetite loss. Chest radiography revealed bilateral infiltration shadows compatible with COVID-19 (Figure 2a) but no evident limb edema. A cardiac ultrasound test was not performed because of the risk of infection. The patient’s parameters were as follows: BT, 36.5 °C; oxygen saturation, 81% (room air); LDH, 257 U/L; CRP, 13.77 mg/dL; D-dimer, 4.14 𝜇g/mL; and procalcitonin, 0.39 ng/mL. He was hospitalized as a moderate II COVID-19 severity patient and received mask oxygenation instead of noninvasive positive-pressure ventilation.

High flow nasal oxygen cannula therapy was not administered because of our limited COVID-19 care unit facility. On the hospitalization day, the patient received remdesivir for five days, dexamethasone (6.6 mg per day for 5 days), and tocilizumab (400 mg) once based on our hospital protocol for moderate II COVID-19 severity. The patient gradually recovered and was transferred to the general ward for more than 10 days after COVID-19 onset as he was not considered contagious anymore. The Charlson comorbidity index was 3 (high) and PCR results were not requested as there was no infection risk. Chest radiography revealed decreased bilateral infiltration shadows with peripheral reduced permeability (Figure 2b), the brain natriuretic peptide (BNP) level was 180 pg/mL, and the oxygenation level returned to normal. Due to long-term bed-bound impairment of ADL, bedside rehabilitation was started, but ADL gradually worsened. To recover from malnutrition, the patient’s appetite was restored; however, because of low consciousness, the food and water intakes were not sufficient, leading to fluid therapy (500 ml per day). The patient did not complain of dysphagia, dysgeusia, or ageusia, and a transfer attempt to a supporting hospital was unsuccessful. The patient died on September 17, and his death was considered due to senility; therefore, an autopsy was not performed.

## Discussion

This case report highlights complications regarding after-care recovery from acute COVID-19. The first issue is the fear of SARS-CoV-2 infection to others. Researchers reported that patients’ infectivity without symptoms is already diminished 10 days after COVID-19 onset, suggesting that patients who recover after 10 days from the disease onset can be transferred to support hospitals. Several supporting hospitals were consulted for rehabilitation; however, despite the fact that the patient recovered from superinfection by bacteria after other virus infections and two rounds of PCR gave negative results, the fear of nosocomial infection was sufficient to deny the transfer. Similar to other countries, COVID-19 isolation rooms were limited in Japan. Therefore, the patients were transferred to our general ward, where personal protective equipment (PPE) was not required. COVID-19 requires isolation in a single room and prohibits going out of the room. In this case, the lack of isolation led an ambulant elderly person to become bedridden and malnourished.

The second issue is that COVID-19 can induce long-term malnutrition and multiple organ damage. During COVID-19-related isolation, general rehabilitation is rare because physical therapists refuse to provide rehabilitation in our region due to various reasons. Multiple organ failure is a sequela of COVID-19 [7]. Moreover, COVID-19 can severely impair pulmonary diffusion capacity imaging manifestations even six months and one year later, has been reported as a future risk factor for pulmonary fibrosis, and can cause residual ground-glass opacities, consolidations, reticular and linear opacities, and parenchymal fibrotic bands in the long term [8]. The time course of COVID-19 lung imaging is currently under investigation; however, COVID-19 can also injure neurons connected to the peripheral respiratory receptors [9].

Some reports have shown better results of early rehabilitation with PPE [10]. However, not all institutions are using PPE because rehabilitation with PPE requires tremendous medical resources. Some hospitals, including ours, do not have sufficient physical therapists for both breathing and swallowing training. Therefore, our human medical resources were limited despite guideline recommendations. Patients with COVID-19 require long-term follow-up due to long-term damage to multiple organs, and rehabilitation after recovery from COVID-19 in the elderly is still effective and improves lung function. Specialized personalized rehabilitation for respiratory, physical, and psychological long-term dysfunction caused by COVID-19 is required [11].

In Japan, in April 2021, two consecutive negative PCR results without the occurrence of symptoms for at least 72 hours, at least 10 days from disease onset, were required for discharge. In April 2021, 8 of 17 beds in the COVID-19 ward of our hospital were occupied in a city housing 63,000 people. The first case was suitable for transfer in June 2021. The government-approved supporting hospitals in our city were already full because other elderly (non-COVID-19) patients needed to stay in hospitals because of low ADL, suggesting that they could not leave the hospital. This was considered one of the reasons for delayed rehabilitation. COVID-19 occurs in cycles, acutely increasing maintenance costs for every hospital. Earlier transfer to supporting hospitals may improve ADLs and lower mortality rates through conventional rehabilitation.

COVID-19 induces damage to multiple organs, suggesting a higher risk of death after recovering from COVID-19 [12]. Our second patient died of being senile resulting from frailness, which started during hospitalization. Several studies have reported the increased risk of frailness from similar aspects: 1) Loss of appetite and sarcopenia induced by frailty is defined as declined muscular function in the presence of muscle loss. The COVID-19 pandemic makes it mandatory for elderly people to “stay at home,” leading to frailty [13]. 2) Social distancing and staying home reduce physical activity and increase other unhealthy lifestyle habits such as loneliness or malnutrition [14]. 3) Poor health after COVID-19 is associated with mortality risk that is independent of respiratory function [15]. Moreover, a meta-analysis concluded that frailty was significantly associated with an increased risk of adverse clinical events (all-cause mortality) [16] The Clinical Frailty Scale (CFS) is a quick and reliable screening tool used to evaluate frailty, and regular home exercises are recommended to prevent frailty [17]. Unfortunately, the Charlson comorbidity index and CFS were not commonly used in our hospital.

We could not properly treat malnutrition in the cases described owing to a lack of rehabilitation and monetary resources. To the best of our knowledge, the effect of direct rehabilitation in improving the simple loss of appetite without dysphagia, dysgeusia, or ageusia in COVID-19 is unknown. It is worth considering whether rehabilitation helps restore appetite as sequelae precisely.

## Conclusions

COVID-19-related death appears to include a health risk after recovery. Early active physical therapy is not common in some hospitals, but it should start from the hospitalization day and continue during the isolation period. Researchers still produce inconsistent results regarding the COVID-19 sequelae care study. Evaluation of simple appetite loss is not well investigated. Further studies by researchers should focus on evaluating each study for real-world applicability to improve ignored malnutrition.
